# First genetic maps development and QTL mining in *Ranunculus asiaticus* L. through ddRADseq

**DOI:** 10.3389/fpls.2022.1009206

**Published:** 2022-09-23

**Authors:** Matteo Martina, Alberto Acquadro, Davide Gulino, Fabio Brusco, Mario Rabaglio, Ezio Portis, Sergio Lanteri

**Affiliations:** ^1^ Dipartimento di Scienze Agrarie, Forestali e Alimentari (DISAFA), Plant Genetics and Breeding, University of Torino, Grugliasco, Italy; ^2^ Biancheri Creazioni, Camporosso, Italy

**Keywords:** ornamentals, ddRADseq, linkage maps, genetic markers, anthocyanins, QTLs

## Abstract

Persian Buttercup (*Ranunculus asiaticus* L.; 2x=2n=16; estimated genome size: 7.6Gb) is an ornamental and perennial crop native of Asia Minor and Mediterranean basin, marketed both as cut flower or potted plant. Currently new varieties are developed by selecting plants carrying desirable traits in segregating progenies obtained by controlled mating, which are propagated through rhizomes or micro-propagated *in vitro*. In order to escalate selection efficiency and respond to market requests, more knowledge of buttercup genetics would facilitate the identification of markers associated with loci and genes controlling key ornamental traits, opening the way for molecular assisted breeding programs. Reduced-representation sequencing (RRS) represents a powerful tool for plant genotyping, especially in case of large genomes such as the one of buttercup, and have been applied for the development of high-density genetic maps in several species. We report on the development of the first molecular-genetic maps in *R. asiaticus* based on of a two-way pseudo-testcross strategy. A double digest restriction-site associated DNA (ddRAD) approach was applied for genotyping two F_1_ mapping populations, whose female parents were a genotype of a so called ‘ponpon’ and of a ‘double flower’ varieties, while the common male parental (‘Cipro’) was a genotype producing a simple flower. The ddRAD generated a total of ~2Gb demultiplexed reads, resulting in an average of 8,3M reads per line. The *sstacks* pipeline was applied for the construction of a mock reference genome based on sequencing data, and SNP markers segregating in only one of the parents were retained for map construction by treating the F_1_ population as a backcross. The four parental maps (two of the female parents and two of the common male parent) were aligned with 106 common markers and 8 linkage groups were identified, corresponding to the haploid chromosome number of the species. An average of 586 markers were associated with each parental map, with a marker density ranging from 1 marker/cM to 4.4 markers/cM. The developed maps were used for QTL analysis for flower color, leading to the identification of major QTLs for purple pigmentation. These results contribute to dissect on the genetics of Persian buttercup, enabling the development of new approaches for future varietal development.

## Introduction

Persian Buttercup (*Ranunculus asiaticus* L.; 2x=2n=16; estimated genome size: 7.6Gb - Goepfert, 1974) is an outcrossing ornamental and perennial crop native of Iran, Turkey and Greece. The species is marketed both as cut flowers and potted plant (De Hertogh, 1996). As reported by Berruto et al. (2019), Ranunculus counted for the 0,4% of the total turnover of cut flowers and foliage, with the highest production in Italy (132 million of stem) and 300-350 ha of cultivated surface. Due to its high level of heterozygosity and self-incompatibility, the crossing of selected genotypes originates highly segregant progenies. The development of new varieties is based on the selection within the progenies of plants carrying traits of interested, which are propagated through rhizomes or micro-propagated *in vitro* from meristematic apexes (Beruto et al., 2018). In order to escalate breeding efficiency and respond to market demand, some more knowledge of buttercup genetics is needed in order to enable the development of molecular breeding approaches.

High throughput DNA sequencing methodology (next generation sequencing; NGS) has rapidly evolved over the past 20 years (Slatko et al., 2018), and novel sequencing-associated protocols allow the access to thousands of genomic regions across the genome (Barchi et al., 2012; Víquez-Zamora et al., 2013; Scaglione et al., 2016; Vukosavljev et al., 2016; van Geest et al., 2017). The reduced-representation sequencing (RRS) represents one of most powerful tools for plant genotyping, especially in case of large genomes such as the one *R. asiaticus.* Indeed, RRS has been applied in the development of high-density genetic maps in many species (Acquadro et al., 2017; Feng et al., 2018; Jin et al., 2019; Song et al., 2020; Toppino et al., 2020; Wang et al., 2020; Valentini et al., 2021).

Here we report on the application of a double digest restriction-site associated DNA (ddRAD) approach for genotyping two F_1_ mapping populations and on development of the first molecular-genetic maps in *R. asiaticus.* The strategy adopted was the two-way pseudo-testcross, previously exploited in a number of out-breeding species (Acquadro et al., 2009; Wu et al., 2010; Portis et al., 2012; Gartner et al., 2013; Torello Marinoni et al., 2018; Mariotti et al., 2020; Wang et al., 2022). The two F1 mapping populations shared a common male parental line, and four parental maps were developed (two of the female parents and two of the common male parent), then aligned on the basis of common SNPs markers. The resulting consensus map included eight linkage groups, corresponding to the haploid chromosome number of the species, and represents a background for future mapping of genes and QTLs and application of marker assisted breeding in the species. Besides, we performed QTL analysis for flower color, identifying a major locus affecting flower purple pigmentation.

## Materials and methods

### Mapping populations and DNA extraction

**Figure 1:**
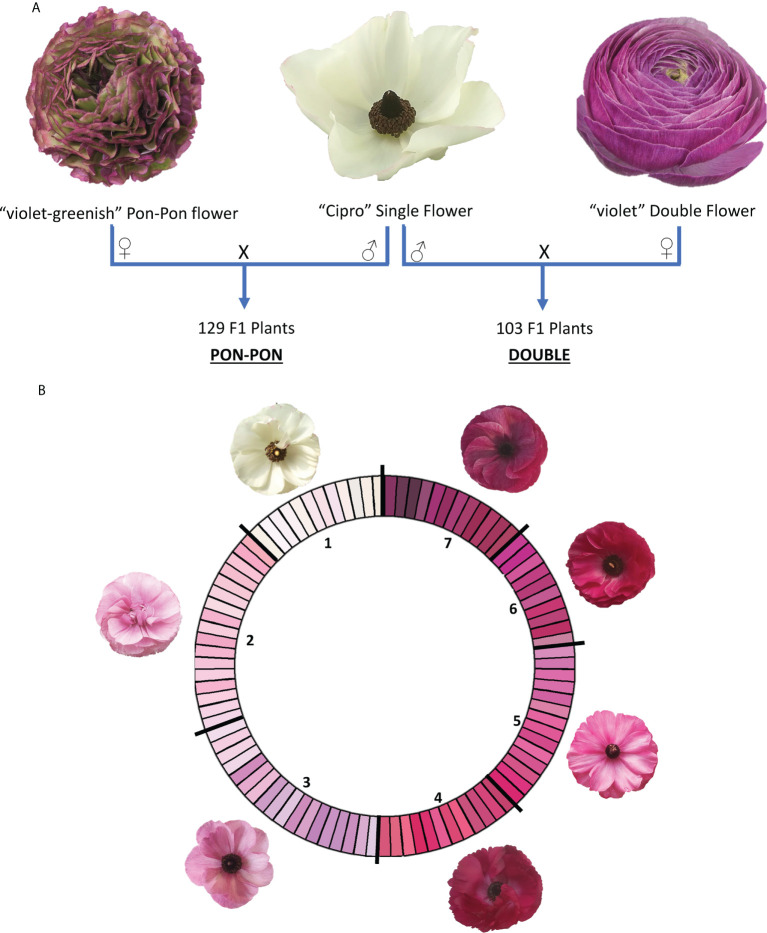
**(A)** Crossing scheme for the development of the two mapping populations (PON-PON and DOUBLE); **(B)** RHS-based color scale used for flower phenotyping. RHS classes were coded according to their intensity in 1-7 scale. 1: white; 2: light blue pink; 3: light violet; 4: medium purple red; 5: dark blue pink; 6: medium purple; 7: dark purple.

Two F_1_ segregating populations were obtained by crossing the male parental genotype ‘Cipro’, producing white flowers with one row of petals typical of the wild-type (called ‘single’ flowers), with two female parental genotypes, of which one producing violet-greenish flowers with wavy margin (called ‘pon pon’ flowers) and one producing violet flowers with multiple rows of petals (called ‘double’ flowers). The progenies were respectively named PON-PON and DOUBLE (**Figure 1A**).

The parental genotypes were selected by Biancheri Creazioni (IM, Italy). The two F_1_ progenies included 129 and 103 plants for the PON-PON and DOUBLE population respectively, and were grown in a green-house located in Camporosso (43.794, 7.632, IM – Italy) for two seasons (2020 and 2021), adopting the cultivation techniques in use for commercial production. In 2020 plants originated from seeds, while in the 2021 were vegetatively propagated through rhizomes obtained at the end of the first season. Genomic DNA was extracted from frozen leaves with the Plant DNA Kit (E.Z.N.A.^®^) following the manufacturer’s instructions. DNA quality was assessed through the NanoDrop™ 2000 spectrophotometer, and the Qubit^®^ 2.0 Fluorometer was used for DNA quantification.

### ddRAD-seq library preparation and sequencing

The ddRAD (double digest restriction-site associated DNA) libraries were produced using a custom protocol with minor modifications proposed by Peterson et al. (2012). Enzyme combination was selected by *in silico* analyzing the draft genome of the related species *Anemone coronaria* L. (Martina et al., 2022), with the aim of selecting a combination predicted to produce ~30k fragments across the provided genome. After fluorometric quantification, the genomic DNA was normalized for uniforming its concentration and 375ng (10ul, ~37ng/ul) were double digested with 2.4U of both *PstI* and *EcoRI* endonucleases (New England BioLabs) in 30µL reaction, supplemented with CutSmart Buffer and incubated at 37°C for 90’, followed by 20’ at 65°C’. Fragmented DNA was purified with 1.5 volumes of AMPureXP beads (Agencourt) and then ligated with 200U of T4 DNA ligase (New England BioLabs) to 2.5pmol of overhang barcoded adapter for rare cut sites and to 5pmol of overhang barcoded adapter for frequent cut sites. Reactions were performed in 50µL volume at 23°C for 60’ and at 20°C for 60’, followed by 20’ at 65°C. Samples were then pooled on multiplexing batches and bead purified as above. For each pool, targeted fragments distribution was collected on the BluePippin instrument (Sage Science Inc.), setting the range of 380 bp – 500 bp. Gel eluted fraction was then PCR amplified with indexed primers using Phusion High-Fidelity PCR Master Mix (New England BioLabs) in a final volume of 50µL, and subjected to the following thermal protocol: [95°C, 3’] - [95°C, 30’’ - 60°C, 30’’ - 72°C, 45’’] x 12 cycles - [72°C, 2’]. Products were purified with 1 volume of AMPureXP beads. The resulting libraries were checked with both Qubit 2.0 Fluorometer (Invitrogen, Carlsbad, CA) and Bioanalyzer DNA assay (Agilent technologies, Santa Clara, CA). Libraries were then PE sequenced with 150 cycles on NovaSeq 6000 instrument following the manufacturer’s instructions (Illumina, San Diego, CA). A low coverage sequencing and a draft assembly of the male parent (‘Cipro’) was obtained as reference for investigating data obtained with ddRADseq.

### Sequence analysis and linkage maps development

Raw reads were demultiplexed using the *process_radtags* pipeline included in Stacks v2.53 (Catchen et al., 2013). Assembly of the short-reads of each sample into matching stacks was performed using the *ustacks* utility included in Stacks v2.53. A set of consensus loci from all the analyzed samples (catalog) was identified by the *cstacks* pipeline, and each sample was matched against the catalog using *sstack* and *tsv2bam* utilities. Sequences for each genotype were mapped to the catalog file using the Burrows-Wheeler Aligner (BWA, v0.7.17) program and the ‘mem’ command with the default parameters (Li and Durbin, 2009). BAM files were processed and used for SNP calling using *Samtools mpileup* (v1.6 - Danecek et al., 2021) with default parameters except for the minimum mapping quality (Q = 20). Markers were named according to the catalog sequence in which they were identified. Each name starts with an S, followed by the catalog sequence number and the position of the SNP in the sequence (i.e *S0004445_269*).

Independent framework linkage maps were constructed for each parent (male and female) from each progeny-based dataset (PON-PON and DOUBLE), using the double pseudo-testcross mapping strategy (Plomion and Durel, 1996) and JoinMap v4.0 (Van Ooijen, 2006). Four separate datasets were assembled: (i) ‘Violet-greenish’ - ‘PP -’; (ii) Cipro I - ‘C-I’ -; (iii) ‘Violet’ - ‘Db’ - and (iv) Cipro II - ‘C-II’. Only SNP markers in testcross configuration (expected segregation ratio of 1:1) were included in the datasets: maternal testcross markers segregating only in ‘PP’ or ‘Db’, and paternal testcross markers segregating only in ‘C-I’ or ‘C-II’.

Genotyping data were quality filtered by removing not segregating markers, markers with >20% missing values, and skewed markers. The similarity of the loci option of JoinMap was used to identify perfectly identical markers (similarity value = 1.000), expected to map to exactly the same position. To reduce the load of calculation effort, only one representative of each group of identical loci was used for mapping. Goodness-of-fit between observed and expected segregation ratios was assessed using the χ^2^ test. Markers fitting a Mendelian pattern closely associated with a χ^2^ value χ^2^ α = 0.1 or with only a minor deviation (χ^2^ α = 0.1< χ^2^ χ^2^ α = 0.01) were used for map construction, provided that their inclusion did not alter the local marker order. Loci suffering from significant segregation distortion (χ^2^ > χ^2^ α = 0.01) were excluded.

For all maps, LGs were established based on a threshold logarithm of odds (LOD) ratio >4. To determine marker order within a linkage group (LG), the JoinMap parameters were set at Rec = 0.40, LOD = 1.0 and Jump = 5. Map distances were converted to centiMorgans (cM) using the Kosambi mapping function (Kosambi, 1943). Linkage maps were drawn using MapChart 2.2 software (Voorrips, 2002), and markers deviating in their segregation only marginally from the expected Mendelian ratio are presented with one (χ^2^ α = 0.1 < χ^2^ χ^2^ α = 0.05) or two (χ^2^ α = 0.05 < χ^2^ χ^2^ α = 0.01) asterisks. LG of the female and male maps were respectively named PP_01 to PP_08 and CI_01 to CI_08 for the ones present in the PON-PON dataset, and Db_01 to Db_08 and CII_01 to CII_08 for the ones present in the DOUBLE dataset (**Supplemental Figure 1** and **Supplemental Table 1**).

### Flower color assessment

The first blooming flower of market quality of each genotype was phenotyped in both seasons (2020 and 2021), as representative of the genetic potential of each individual. The main color of the internal (Main Interior Color - MiC) and external (Main Exterior Color - MeC) layer of the petals were scored following RHS classification (**Figure 1B**).

### Statistical analyses and QTL detection

Statistical analyses were performed with R software (Team, 2016). A conventional analysis of variance was applied to estimate genotype and environment effects based on the linear model Yij = μ + gi + bj + eij, where μ, g, b and e represent the mean, the genotypic effect, the block effect and the error respectively. Correlations between traits were estimated using the Spearman coefficient, and normality, kurtosis and skewness were assessed with the Shapiro–Wilks test (α = 0.05). QTL detection was performed by considering each season independently and was based on the newly developed map using MQM mapping, as implemented in MapQTL v5 software (Van Ooijen, 2004). QTLs were initially identified using interval mapping. LOD thresholds for declaring a QTL to be significant at the 5% genome-wide probability level were established empirically by applying 1000 permutations per trait (Doerge and Churchill, 1996). After permutation analysis, linked marker per putative QTL was treated as a co-factor in the approximate multiple QTL model. Co-factor selection and MQM analysis were repeated until no new QTL could be identified. Additive effect, as well as the percentage of the phenotypic variation (PVE) explained by each QTL, were obtained from the final multiple QTL model. Individual QTLs were prefixed by the parental line abbreviation (‘PP’, ‘C-I’, ‘C-II’, or ‘Db’), followed by a trait abbreviation, the relevant chromosome designation, while the season was followed by a letter when there were more than one QTL for the season. Confidence interval of the QTL was calculated by considering 0.5Mb upstream and downstream the marker identified at the QTL.

## Results and discussion

### Sequencing and linkage map construction

The ddRAD approach generated a total of ~2Gb demultiplexed reads, and an average of 8,3M reads per genotype (detailed info about the raw data per individual can be found in Supplemental **Table 1**). Cleaned reads with quality scores >30 were mapped against the catalog obtained through the *stacks* pipeline (see Materials and Methods). Before filtering, ~578k polimorphisms where identified. However, after filtering at DP>15, this number was lowered to ~22k SNPs and 790 indels. Only markers heterozygous in a single parent were retained for map construction, by treating the F1 population as a backcross. For each of the parental lines, skewed markers, showing highly significant distortion from the expected 1:1 ratio, or markers with identical segregation patterns were excluded from further map construction steps (**Supplemental Table 1**).

**Table 1:** Statistics of the four linkage maps.

		LG 1	LG 2	LG 3	LG 4	LG 5	LG 6	LG 7	LG 8	Average	Total
**PonPon (PP)**	*Size*	112.0	130.7	97.4	113.4	102.9	80.5	110.8	82.2	103.7	829.8
	*N° of markers*	93	83	63	82	74	58	37	78	71	568
	*Marker Density*	1.2	1.6	1.5	1.4	1.4	1.4	3.0	1.1	1.6	
	*Gaps (> 5cM)*	3	3	4	1	2	2	8	0	3	23
**Cipro I (C-I)**	*Size*	199.1	201.4	189.5	173.6	156.1	123.7	147.8	114.6	163.2	1305.8
	*N° of markers*	102	75	64	72	74	55	49	65	70	556
	*Marker Density*	2.0	2.7	3.0	2.4	2.1	2.2	3.0	1.8	2.4	
	*Gaps (> 5cM)*	5	9	13	6	4	9	11	3	8	60
**Cipro II (C-II)**	*Size*	226.7	160.2	116.6	132.6	151.4	149.0	127.0	118.8	147.8	1182.2
	*N° of markers*	152	98	113	71	49	73	50	74	85	680
	*Marker Density*	1.5	1.6	1.0	1.9	3.1	2.0	2.5	1.6	1.9	
	*Gaps (> 5cM)*	6	4	3	3	9	6	9	2	5	42
**Double (Db)**	*Size*	169.1	145.6	102.5	161.4	151.1	106.9	105.4	117.6	132.5	1059.7
	*N° of markers*	68	33	65	112	68	74	46	74	68	540
	*Marker Density*	2.5	4.4	1.6	1.4	2.2	1.4	2.3	1.6	2.2	
	*Gaps (> 5cM)*	9	12	4	4	9	2	5	6	6	51

**Figure 2:**
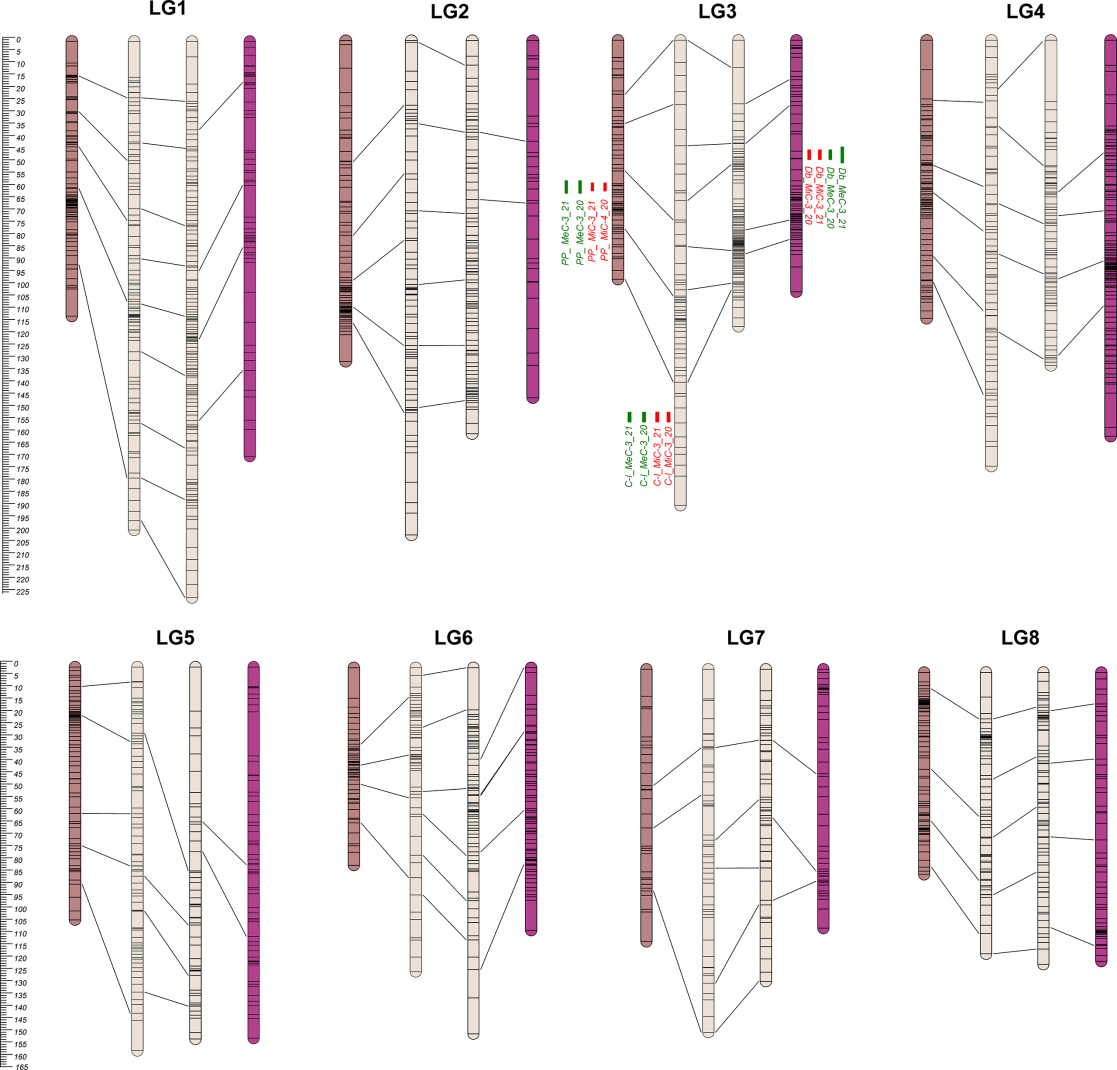
From the left to the right: PP, C-I, C-II, and Db Linkage Groups. Eight LGs (corresponding to the haploid chromosome number of the species) were identified within each parental map. C-I map was the bigger one, covering 1,305.8 cM, while the PP map was the smaller one with 829.8 cM (**Table 1**). QTL regions are reported as green (MeC) and red (MiC) bars on the right side of the associated LGs.

Overall, 3,376 single nucleotide polymorphisms (SNPs) were identified as segregating in the PONPON population, of which 1,607 and 1,769 were used as input in JoinMap for the development of Cipro (C-I) and PonPon (PP) maps, respectively. In C-I, 732 markers were excluded by the JoinMap pipeline being co-segregating (i.e. markers showing the same segregation pattern), leading to an overall number of 875 markers used for map construction. Analogously, in PP, 796 SNPs were co-segregating, while 973 were used for map construction. A further stringent selection was applied by considering only markers grouped at LOD >4, and a set of 568 SNPs in PP, and 556 in C-I were identified as suitable for map development. The generated PP map spanned 829 cM over 8 LGs (corresponding to the haploid chromosome number of the species) ranging from 80.5 to 130.7 cM, with a marker density between 1.1 and 3 cM, and 23 gaps bigger than 5 cM. The LG7 harbored the highest number of gaps (8), while only one gap was present in LG4. The C-I map spanned 1305.8 cM across 8 LGs, ranging between 123.7 and 201.4cM and with a marker density between 1.8 and 3 cM. In this map a higher number of gaps (>5 cM) was present (60), with LG3 and LG7 including 13 and 11 gaps respectively, and LG8 harboring the lower number of gaps (3). The LGs of the two maps were aligned on the basis of 35 common markers, as shown in **Figure 2**.

Overall, 4,098 SNPs were identified in the DOUBLE population, of which 2,344 and 1,754 segregating in Cipro (C-II) and Double (Db) maps respectively. In C-II, 1,211 co-segregating markers were discarded and 1,113 SNPs used for map construction. For Db, 919 SNPs were co-segregating, leading to 835 mappable markers. A further stringent selection was applied by considering only markers grouped at LOD >4, identifying 680 loci for C-II, and 540 for Db maps development. A genetic map with an overall dimension of 829 cM was generated for Db, identifying 8 LGs ranging from 102.5 and 169.1 cM with a marker density between 1.4 and 4.4, and 51 gaps bigger than 5 cM. The highest number of gaps (12) was scored on LG2, while only two gaps were present on LG6. The 8 LGs developed for the C-II map covered 1182.2 cM, ranging between 116.6 and 226.7 cM with a marker density between 1 and 3.1 cM. Forty-two gaps were identified across LGs, with LG5 and LG7 presenting 9 gaps, and LG8 harboring only 2 gaps. Each LGs of the two maps were aligned using 27 common markers, as shown in **Figure 2** and **Table 1**.

The 8 LGs of the two maps of the male parental genotype (C-I and C-II) were aligned by means of 44 markers. Furthermore, the four maps were aligned on the basis of 106 common markers of which 7 markers were common across PP, C-I and C-II, while one was common among the four maps. A clustering of markers was observed in some LGs, and in most cases was located in the central part of a LG. This clustering might correspond to the centromeric regions (Keim et al., 1990; Reiter et al., 1992; Tanksley et al., 1992; Vallejos et al., 1992; Ott et al., 2011), in which a reduced recombination usually takes place (Vincenten et al., 2015; Zafar et al., 2017; Acquadro et al., 2020).

An average number of 586 markers was included in each parental map, with a marker density ranging from 1 marker/cM to 4.4 markers/cM. Assuming an expected genome size of 7.6Gb (Goepfert, 1974), the average mapping length of ~1093 cM was used for estimating the physical dimension of a cM, which corresponds to a physical distance of about 7Mb. The average LG dimension ranged from 829.8 cM (PP) to 1305.8 cM (C-I).Both the male parent maps (C-I and C-II) were larger than the ones of the female parents (PP and Db). As previously reported (Lenormand and Dutheil, 2005), differences between paternal and maternal linkage maps could be associated with heterochiasmy (i.e., the presence of different crossover frequencies in male and female meiosis). Differential recombination rates between the sexes have been reported in several plant species, and attributed to processes such as sexually antagonistic selection acting on coding and regulatory genetic elements, female meiotic drive as well as selection during the haploid phase of the life cycle (Sardell and Kirkpatrick, 2020). The latter has been associated to the reproductive system (selfing or outcrossing) characterizing the species (Giraut et al., 2011; Mariotti et al., 2020), although even in closely related species sharing the same reproduction pattern, such as *Sinapis alba* (Nelson et al., 2013), *Brassica oleracea* (Kearsey et al., 1996) and *Brassica napus* (Kelly et al., 1997), contrasting results have been reported.

### Phenotypic variation, traits correlation and QTL identification

**Table 2** reports a summary of the phenotypic performance for main color of the internal (MiC) and external (MeC) layer of the petals in the parental genotypes and F1 population, together with skewness and kurtosis of the traits. The male parent ‘Cipro’ did not present anthocyanin content in both petal layers. Conversely, the ‘Double’ genotype produced violet flowers, and PonPon violet- greenish petals. While the MiC trait appears to be stable in the two seasons in the parental lines, MeC showed a certain level of variability in the PP parent. In the F1 populations transgressive segregation was observed in respect to both maternal parents, except for MeC in the DOUBLE population.

**Table 2:** List of the traits analyzed and their code, means, standard deviations (SD), overall statistics, coefficients of variation (cv), and transgressive genotypes.

Population	PON-PON				DOUBLE			
**Trait**	*MiC_20*	*MiC_21*	*MeC_20*	*MeC_21*	*MiC_20*	*MiC_21*	*MeC_20*	*MeC_21*
**Trait code**	*PP_MiC_20*	*PP_MiC_21*	*PP_MeC_20*	*PP_MeC_21*	*Db_MiC_20*	*Db_MiC_21*	*Db_MeC_20*	*Db_MeC_21*
**PP/Db mean**	6	6	7	4	5	5	7	7
**C mean**	1	1	1	1	1	1	1	1
**F1 mean**	2.7	3.4	3.0	3.7	3.4	3.6	4.0	4.2
**± SD**	1.7	2.0	1.9	2.2	1.7	1.7	2.0	2.1
**Cv**	0.6	0.6	0.6	0.6	0.5	0.5	0.5	0.5
**Shapiro-Wilks**	0.80	0.80	0.80	0.78	0.85	0.85	0.79	0.77
**Skewness**	-1.13	-1.61	-1.41	-1.69	-0.18	-0.42	-0.53	-0.63
**SE**	0.43	0.43	0.43	0.43	0.24	0.24	0.24	0.24
**Kurtosis**	0.48	-0.11	0.19	-0.22	-1.20	-1.01	-1.32	-1.26
**SE**	0.22	0.21	0.22	0.21	0.48	0.47	0.48	0.47
**Transgressive respect PP/Db**	4	45	0	58	17	15	0	0
**Transgressive respect C**	0	0	0	0	0	0	0	0

Ponpon parent (PP) and double parent (Db) mean are reported, as well as the Cipro mean (C mean) and the average of the corresponding F1 population (F1 mean). F1 population’s distribution histograms for the investigated traits can be found in in **Supplemental Figure 2**.

Significant inter-trait correlations were detected within and across growing seasons (**Table 3**), albeit detecting a certain level of variability introduced by the environment. The least correlated traits were *DB_MiC_20* and *DB_MeC_21* (+0.70), while the most highly correlated were *PP_MiC_21* and *PP_MeC_21* (+0.94).

**Table 3:** Inter-trait Spearman correlations assessed in the mapping populations.

	PP_MiC_20	PP_MiC_21	PP_MeC_20	PP_MeC_21
PP_MiC_20	–			
PP_MiC_21	0.76	–		
PP_MeC_20	0.90	0.81	–	
PP_MeC_21	0.77	0.94	0.84	–
	Db_MiC_20	Db_MiC_21	Db_MeC_20	Db_MeC_21
Db_MiC_20	–			
Db_MiC_21	0.82	–		
Db_MeC_20	0.88	0.81	–	
Db_MeC_21	0.70	0.88	0.86	–

In Season 2020, the plants originated from seeds were less vigorous in respect to plants originated from rhizomes in season 2021. The flower color was limitedly affected by the environment traits in the two seasons. However, in Season 2020 the PP population distribution appeared to be leptokurtic, while in the Season 2021 the distribution was platykurtic. Such differences did not appear in the DOUBLE population. Transgressive genotypes always deviate towards the more pigmented parent (PP and Db), with petals exceeding the parental lines in pigment accumulation. Furthermore, in some cases the petal color appeared to be not uniform and presented mottling, suggesting the involvement of regulatory elements and epigenetic mechanisms, as reported in literature (Koseki et al., 2005; Le Maitre et al., 2019; Wang et al., 2019; Zheng et al., 2021).

The genetic basis of variation in quantitative traits can traditionally be resolved by the QTL approach, which partitions the variation into distinct genomic regions defined by a linkage map (Paterson et al., 1988). One of the most important issues to determine is the stability of the trait and how stable the expression of the various loci is by repeating the phenotypic evaluation over time and/or space. QTL detection was performed considering each season independently and was based on the newly developed map using MQM mapping (see Materials and Methods). LOD score, percentage of variance explained (PVE), and confidence interval (CI) related to QTLs, are described in **Table 3**. QTL analyses on all traits and environments yielded a total of 12 major (PVE values >10), located on LG3 (see **Figure 2** and **Table 4**). In both female parents (PP and Db), 8 major QTLs were located in the same scaffold (*S0004445)* for both MiC and MeC. On the other hand, QTLs were identified only in the male parental map from the PON-PON population (C-I), both for MiC and MeC. Our results highlight a very high correlation between the external (MeC) and internal (MiC) pigmentation of the flower (**Table 3**), suggesting that the same genomic regions affect both traits. Thus, MiC and MeC appear to be controlled by common major QTLs located on LG3, justifying a PVE ranging from 31.7 to 84%, with a LOD ranging from 11.23 to 50.6.

**Table 4:** QTL detected in the mapping population.

Map	Trait	Year	Name	Group	Pos.	Locus	LOD	CI	PVE	Add.	GW
PP	MiC	20	*PP_MiC-3_20*	LG3	60.8	S0004445_269	21.74	58.8-61.3	48.3	2.5	18
		21	*PP_MiC-3_21*	LG3	60.8	S0004445_269	38.66	58.8-61.3	67.4	3.4	23
	MeC	20	*PP_MeC-3_20*	LG3	60.8	S0004445_269	29.2	58.8-61.3	49.4	2.7	19.4
		21	*PP_MeC-3_21*	LG3	60.8	S0004445_269	46.1	58.8-61.3	71.9	3.9	25.8
C-I	MiC	20	*C-I_MiC-3_20*	LG3	155.9	S0038600_63	23.32	155.38-156.38	57.9	-2.7	18
		21	*C-I_MiC-3_21*	LG3	155.9	S0038600_63	43.45	155.38-156.38	79.3	-3.7	23.1
	MeC	20	*C-I_MeC-3_20*	LG3	155.9	S0038600_63	28.81	155.38-156.38	65.7	-3.1	19.6
		21	*C-I_MeC-3_21*	LG3	155.9	S0038600_63	50.6	155.38-156.38	84	-4.1	26
Db	MiC	20	*Db_MiC-3_20*	LG3	48.3	S0004445_68	11.23	47.565-48.796	31.7	-2.0	9.3
		21	*Db_MiC-3_21*	LG3	48.3	S0004445_68	15.62	47.565-48.796	45.1	-2.4	10.1
	MeC	20	*Db_MeC-3_20*	LG3	48.3	S0004445_68	22.37	47.565-48.796	59	-3.2	14.8
		21	*Db_MeC-3_21*	LG3	48.3	S0004445_68	21.41	47.565-48.796	58.9	-3.3	18.7

For each trait the location (LG and position), the associated marker, the LOD, confidence interval (CI), percentage of explained variation ( PVE), additive effect (Add.), and the genome-wide thresholds (GW) at p = 0.05 (as determined from 1000 permutations) are indicated.

To the best of our knowledge, this is the first report on QTLs detection in Persian buttercup. The identified QTLsshould be further on investigated and, once validated on a large collection of breeding material, the developed molecular markers might be applied for molecular assisted breeding.

## Conclusions

Genome size varies greatly across the flowering plants and has played an important role in shaping their evolution. *Ranunculus asiaticus* harbors a very large genome size of about 7Gb.Mainly as a consequence of its large genome size, the development of genomic tools in the species has been to date very limited. We have applied for the first time in Persian buttercup, a reduced -representation sequencing (RRS) approach with the goal to develop its first genetic maps. Based on the two-way pseudo test cross strategy in two F1 populations, sharing the same male parent, we constructed maps which were used for positioning the first QTLs affecting the flower anthocyanin pigmentation. Our results lay the foundations for future genetic and genomic studies and provide a framework for implementing more targeted breeding programs in *R. asiaticus*.

## Data availability statement

Sequencing data used in this study are openly available in the NCBI database (PRJNA876570 - https://www.ncbi.nlm.nih.gov/bioproject/PRJNA876570).

## Author contributions

Conceptualization: SL, EP, AA MR, FB and MM; methodology: SL, EP, AA MR, and MM; software: AA, EP and MM; validation, MM, DG and EP; formal analysis: MM, DG and EP and AA; investigation: EP, MM, AA; resources: MR, FB, and DG; data curation: MM and EP; writing—original draft preparation: MM, AA, and EP; writing—review and editing: SL; visualization: MR, FB, and DG; supervision, EP and SL. All authors have read and agreed to the published version of the manuscript.

## Funding

This research was partially funded by Biancheri Creazioni (Camporosso, Italy).

## Conflict of interest

The authors declare that the research was conducted in the absence of any commercial or financial relationships that could be construed as a potential conflict of interest.

## Publisher’s note

All claims expressed in this article are solely those of the authors and do not necessarily represent those of their affiliated organizations, or those of the publisher, the editors and the reviewers. Any product that may be evaluated in this article, or claim that may be made by its manufacturer, is not guaranteed or endorsed by the publisher.

## Supplementary material

Supplementary Figure 1 Linkage Groups obtained after analysis, together with markers name.

Supplementary Figure 2 Distribution of the investigated traits in the two populations.
